# New Experimental Models of Diabetic Nephropathy in Mice Models of Type 2 Diabetes: Efforts to Replicate Human Nephropathy

**DOI:** 10.1155/2012/616313

**Published:** 2012-02-08

**Authors:** María José Soler, Marta Riera, Daniel Batlle

**Affiliations:** ^1^Department of Nephrology, Hospital del Mar-IMIM, 08010 Barcelona, Spain; ^2^Division of Nephrology and Hypertension, Department of Medicine, Feinberg School of Medicine, Northwestern University, Chicago, IL, USA

## Abstract

Diabetic nephropathy (DN) is the leading cause of end-stage renal disease. The use of experimental models of DN has provided valuable information regarding many aspects of DN, including pathophysiology, progression, implicated genes, and new therapeutic strategies. A large number of mouse models of diabetes have been identified and their kidney disease was characterized to various degrees. Most experimental models of type 2 DN are helpful in studying early stages of DN, but these models have not been able to reproduce the characteristic features of more advanced DN in humans such as nodules in the glomerular tuft or glomerulosclerosis. The generation of new experimental models of DN created by crossing, knockdown, or knockin of genes continues to provide improved tools for studying DN. These models provide an opportunity to search for new mechanisms involving the development of DN, but their shortcomings should be recognized as well. Moreover, it is important to recognize that the genetic background has a substantial effect on the susceptibility to diabetes and kidney disease development in the various models of diabetes.

## 1. Introduction

Diabetic nephropathy is one of the major long-term microvascular complications and is the major cause of morbidity and premature mortality in individuals with type 2 diabetes mellitus. End-stage renal disease (ESRD) in patients with type 2 diabetes has increased dramatically worldwide during the last few decades, and diabetes is associated with worse survival among patients undergoing dialysis [1–3]. The pathogenesis of diabetic nephropathy includes both metabolic and hemodynamic factors [4]. A large number of candidate genes have been analyzed, both regarding initiation and progression but still are weak predictors of nephropathy in patients with type 2 diabetes. Experimental models of type 2 diabetes with nephropathy may offer a key to a better understanding of this complication in a multifactorial disease such as type 2 diabetes. Several experimental models have been developed to try to mimic human type 2 diabetes [5]. It seems that the ideal animal model for DN research in type 2 diabetes should have human-like kidney anatomy, hemodynamics, and physiology; develop the human type 2 characteristics and complications; allow studies in chronic stable DN, and allow measurement of relevant hemodynamic and biochemical parameters. However, it is difficult to generate a single-mouse model that recapitulates all of the features of established human diabetic nephropathy [6]. In 2003, the lack of reliable mouse models for studying severe DN prompted the National Institute of Diabetes and Digestive and Kidney Diseases (NIDDK) to fund a consortium for the development and phenotyping of new diabetic mouse models that would resemble human DN more closely. 

The Animal Models of Diabetic Complications Consortium (AMDCC) defined criteria for validating murine models of human diabetes and diabetic complications: >50% decline in GFR over the lifetime of the animal, >10-fold increase in albuminuria compared with controls for that strain at the same age and gender, and pathology of kidneys (advanced mesangial matrix expansion, ± nodular sclerosis mesangiolysis, any degree of arteriolar hyalinosis, glomerular basement membrane thickening by >50% over baseline and tubulointerstitial fibrosis) [7]. The purpose of this paper is to summarize the features of the well-established and newer models of experimental nephropathy and their similarities to DN in patients with type 2 diabetes. There are many similarities in kidney disease findings in animal models of type 1 and type 2 diabetes, like in humans, which will not be discussed in the paper. Exacerbation of diabetic kidney disease by manipulations of the renin-angiotensin system such as renin overexpression [8], knockout of the bradykinin B2 receptor [7], or decorin deficiency [9] that have been studied so far in models of type 1 diabetes will not be discussed here, but the approaches are likely to be useful for type 2 diabetes as well.

## 2. Diabetic Nephropathy in Humans

**Figure 1:**
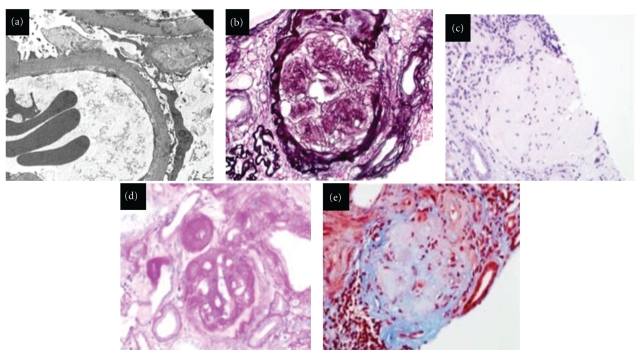
Established DN in humans: (a) glomerular basement membrane thickening by electron microscope. (b) Glomerular nodules in DN with intensively positive staining by methenamine silver. Nodular glomerulosclerosis: (c) hematoxilin eosine staining, (d) periodic acid-schiff stain demonstrates the mesangial nodules and esclerosis glomerular. (e) nodular diabetic glomerulosclerosis by Masson's trichrome staining, (Magnification ×400) in collaboration with Dr. Javier Gimeno.

In 1959, Gellman et al. [10] first reported findings in renal biopsies from patients with diabetic kidney disease and their clinical correlates. Before this was studied, the renal pathology in patients with diabetes mellitus had only been described systematically from autopsy findings. The initial changes of DN are glomerular hypertrophy, mild mesangial expansion (matrix), and thickening of the glomerular capillary walls (Figure 1). These changes are more evident by electron microscopy. When the disease progresses, there is also an increase in mesangial expansion; this mesangial matrix increase is defined as an increase in extracellular material in the mesangium such that the width of the interspace exceeds two mesangial cell nuclei in at least two glomerular lobules. As the disease progresses, there may be formation of nodules in the glomerular tuft. The nodules have variable size in a same glomerulus and affect some portions of the glomeruli, a pattern referred to as nodular diabetic glomerulosclerosis or *Kimmelstiel-Wilson nodules *(Figure 1). The presence of these nodules, large and small, some laminates, with variable size and distribution in the glomeruli, are “virtually” pathognomonic of DN, although similar nodules have been described as “idiopathic” in nondiabetic patients [11–13]. In DN, the glomeruli present increase of the mesangial intercapillary matrix, with progressive thickening of capillary walls and later evolution to global glomerulosclerosis.

Other two glomerular lesions, called “insudative lesions” (similar to arteriolar hyalinosis), are *capsular drop* and *glomerular hyalinosis*. The first is a homogenous hyaline deposit, in the Bowman's capsule. Usually this deposit is rounded or elongated, and its presence is highly suggestive of DN [14].

Glomerular hyalinosis is caused by a formation of a hyalin cap or a fibrin cap by accumulated plasma components in the peripheral segments of the tuft. In many typical DN cases, microaneurysms, produced by mesangiolysis, are evident. In tubules, there are nonspecific changes: reabsorption of protein droplets, interstitial fibrosis, and tubular atrophy. In vessels, usually there are notorious changes; the most characteristic lesion is intimal hyaline thickening of arterioles. Arteriolar lesions may involve any arteriole. Demonstration of arteriolar hyalinosis in both the afferent and efferent arterioles is virtually pathognomic of DN. Intimal fibrosis of the arteries is typical of DN, but this feature is not pathognomic because intimal fibrosis occurs in other diseases as well [15].

## 3. Classical Models of Nephropathy in Type 2 Diabetes

### 3.1. LepR^db/^LepR^db^ (db/db)

**Figure 2:**
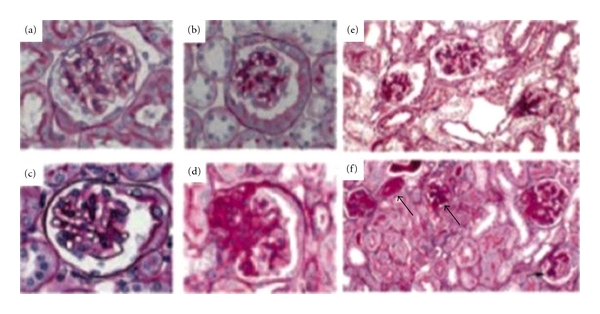
Glomerular histopathology in eNOS^−/−^ db/db mice. Representative glomerular lesions of diabetic mouse kidneys at 24–26 wk: control (a); db/db (b and e); eNOS^−/−^ (c) eNOS^−/−^ db/db (d and f). (f) Arteriolar hyalinosis (big arrow) and early nodular glomerulosclerosis (small arrow) in eNOS^−/−^ db/db mice (periodic acid-Schiff). Reprinted with permission from the Journal of the American Society of Nephrology [25].

The first described mutation in mice that resembled diabetes mellitus in humans occurred in an inbred mouse strain (C57BL/Ks) in 1966 [16]. The diabetic mutant is similar to the obese mutant in appearance [17], and the diabetic gene is transmitted as an autosomal recessive trait resulting from a mutation of the leptin receptor [16], leading to abnormal splicing and defective signaling of the adipocyte-derived hormone leptin [18]. Kidney function in mice with the db/db mutation on the C57BL/KS background has been intensively investigated and exhibits some features similar to early human diabetic nephropathy, class I to III. This model is widely used as a model of kidney disease and morbid obesity in type 2 diabetes (Figure 2). DN in the C57BL/KsJ(db/db) mouse is initially expressed as increased urinary albumin excretion at the age of 8 weeks without evidence of glomerular lesions by light microscopy [19].

Kidney hypertrophy has been noted in *db/db *mice at the age of 16 wk [20–22]. Glomerular hypertrophy has been measured by a digital planimeter using standard measurements of glomerular tuft areas at various ages of the *db/db *and *db/m *mice [23, 24]. Glomerular surface area was increased at 8 wk of age and remained increased at 25 wk of age. The time course of mesangial matrix expansion was described by Cohen et al. [23]. At 12 wk of age and after 4–6 wk of hyperglycemia, a twofold increase in mesangial matrix was noted. After 16 wk of age, a consistent threefold increase in mesangial matrix expansion was reported. At 5-6 mo of age, diabetic mice had larger glomeruli with increased mesangial matrix by periodic acid-Schiff (PAS) staining (Figure 2). By 18–20 mo of age, the mesangial matrix and glomerular enlargement became more pronounced and thickening of the glomerular basement membrane (GBM) was notable. In the oldest diabetic mice studied (16–22 mo of age), strikingly large subepithelial nodular densities were observed along with foot process fusion [24]. Albuminuria according to some studies is not very progressive in this model [24]. However, in the C57 background, which is generally resistant to kidney disease, albumin-creatinine ratio, in our experience, increases substantially as the animals age from 8 to 32 weeks of age [19].

### 3.2. Lep^ob/^Lep^ob^

The ob/ob recessive obese mouse carries a mutation in leptin, the ligand for the leptin receptor [26]. The Lep^ob^ mutation exists in different strains such as DBA2/J and C57BL/6J and FVB strains [27]. Renal structure and function in C57BL/6J ob/ob mice is said to be relatively mild. Chua et al. observed increased mesangial matrix expansion in FVB ob mice [27]. This model is not often used currently. 

### 3.3. New Zealand Obese Mice

New Zealand obese (NZO) mice are an inbred strain originally selected in New Zealand for polygenic obesity, and it is known to have obesity/diabetes as a result of QTLs on chromosomes 1, 2, 4, 5, 6, 7, 11, 12, 13, 15, 17, and 18 [28–30]. Mice are also prone to autoimmune disease as the kidneys exhibit light microscopic features that resemble lupus nephritis, not just diabetes lesions. The changes include glomerular proliferation, mesangial deposits, mild basement membrane thickening, and glomerulosclerosis [31, 32]. Eosinophilic nodules may be seen in some glomeruli, with occasional hyalinization of the glomerular arterioles and healing arteriolar inflammation [31, 32]. 

### 3.4. Agouti Mutation

The *agouti *gene is expressed during the hair-growth cycle in neonatal skin where it functions as a paracrine regulator of pigmentation [33, 34]. The secreted agouti protein antagonizes the binding of the a-melanocyte-stimulating hormone to its receptor (melanocortin 1 receptor) on the surface of hair bulb melanocytes, causing alterations in intracellular cAMP levels [33, 34]. Widespread, ectopic expression of the mouse *agouti *gene is central to the yellow obese phenotype, as demonstrated by the molecular cloning of several dominant *agouti *mutations and the ubiquitous expression of the wildtype *agouti *gene in transgenic mice. The hypothalamus and adipose tissue are biologically active target sites for *agouti *in the yellow obese mutant lines [33, 34]. Reports of albuminuria in diabetic KK-Ay mice suggest this mutation may be useful for the study of nephropathy. Moreover, the pathological changes in glomeruli from KK-Ay/TA mice are consistent with those in the early stage of human diabetic nephropathy [35]. This animal model of type 2 diabetes has, therefore, been proposed to examine the early stages of diabetic nephropathy [35]. It should be noted, however, that with aging diabetic KK-Ay mice develop spontaneous hydronephrosis, extremely dilated pelvis, and thinned atrophied parenchyma. [36]. 

### 3.5. High-Fat Diet

A high-fat diet (HFD) provides a commonly used approach to induce obesity and insulin resistance in C57BL6 mice [37] and is also useful to study accelerated atherosclerosis associated with diabetes [38, 39]. Surwit et al. showed that genetic differences in the metabolic response to fat are more important in the development of obesity and diabetes than the increased caloric content of a high-fat diet [37]. HFD to C57BL/6 mice induces major systemic alterations compatible to human metabolic syndrome, including obesity, hyperglycemia, hyperinsulinemia, hypertriglyceridemia, and hypertension [40]. After the onset of metabolic syndrome, mice on an HFD developed increased UAE and glomerular lesions with the accumulation of extracellular matrix protein. Furthermore, renal pathophysiological alterations, including lipid accumulation, macrophage infiltration, increased oxidative stress, and impaired sodium handling were observed in the mice on an HFD [40].

## 4. New Models of DN

Previous animal models of diabetic kidney disease have manifested albuminuria and early renal pathologic changes such as glomerular basement thickening and mesangial expansion but with only minimal or inconsistent expression of other characteristic histopathologic features such as arteriolar hyalinosis and nodular glomerulosclerosis. The failure of previous models to manifest these lesions prompts the development of new models of DN (Table 1). 

### 4.1. Db/db eNOS^−/−^

Targeting Nos3, the gene encoding eNOS, or endothelial nitric oxide synthase was found to result in nephropathic changes in mouse models of type 1 and type 2 diabetes that mimic many aspects of human disease due to inhibition of nitric oxide formation [25]. C56BL/KS Db/db eNOS^−/−^ mice developed striking albuminuria and characteristic pathologic changes of DN such as mesangiolysis, microaneurysms, and increased mesangial matrix expansion. In addition, nodular lesions (nodular glomerulosclerosis) and globally sclerotic glomeruli (diffuse glomerulosclerosis) were reported at 26 weeks [25, 41]. This model also exhibited remarkably decreased GFR on the basis of inulin clearance and serum creatinine measurements [25]. Studies in eNOS^−/−^ mice demonstrated that this KO model develops moderate systemic hypertension. The increase in SBP was slightly greater in diabetic db/db eNOS^−/−^ mice, although the results did not reach statistical significance [25]. These studies demonstrated that db/db eNOS^−/−^ mice exhibit significant albuminuria and glomerular pathology that parallel the later phase of DN in patients with type 2 diabetes including arteriolar hyalinosis, mesangial expansion, thickening of GBM, and focal segmental and early nodular glomerulosclerosis [25, 41] (Figure 2). This model should prove useful for studying the role of endothelial dysfunction in development of DN and in facilitating the development of new diagnostic and therapeutic interventions.

### 4.2. NONcNZO10/LtJ

A NONcNZO10/LtJ is an inbred congenic strain derived from a cross between the Nonobese Nondiabetic (NON/LtJ) strain and the New Zealand Obese (NZO/HlLt) mouse, which provides a model of polygenic type 2 diabetes [29, 30, 42]. After approximately 8 mo of age, these mice also develop significant and progressively increasing albuminuria, with urine albumin-creatinine ratios >1000 *μ*g/mg after 1 yr. Glomerular histopathology is impressively abnormal. In addition to glomerulosclerosis, however, there were features that are atypical of diabetic nephropathy. These included intraglomerular capillary thrombi and lipid deposition, nephritis, and evidence of Ig deposition. These features suggest that this may not be a good model for studying DN [29, 30, 42].

### 4.3. BTBR^ob/ob^

Clee et al. characterized a mouse model of insulin resistance that develops in the progeny of the BTBR (black and tan, brachyuric) mouse strain crossed with C57BL/6 mice [43, 44]. BTBR mice are naturally hyperinsulinemic when compared with C57BL/6 mice, and BTBR × C57BL/6 F1 mice are substantially insulin resistant [44, 45]. When the *ob/ob *mutation is placed on a BTBR background, the mice are initially insulin resistant with elevated insulin levels, pancreatic islet hypertrophy, and marked hyperglycemia by 6 weeks of age [46]. 

**Figure 3:**
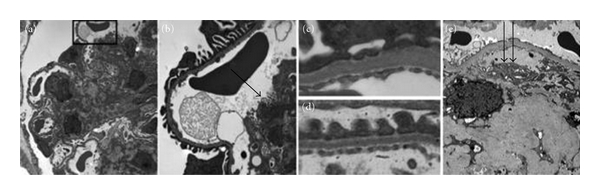
Ultrastructural changes in BTBR ob/ob mice resemble human DN. (a and b) Electron microscopy of glomeruli of 22-week-old BTBR ob/ob mice shows qualitatively good preservation of foot processes overall. There is an increased mesangial matrix and evidence of mesangiolysis with fraying of the mesangial/capillary interface (arrows) in (b). (c and d) Basement membranes are thickened, and there is focal effacement of foot processes in BTBR ob/ob mice (c) when compared with BTBR WT mice (d). There is no evidence of immune deposits. (e) Advanced human DN, occurring after one or more decades of diabetes, also shows marked mesangial matrix accumulation with similar fraying of the mesangial/capillary interface as seen in BTBR ob/ob mice (double arrows). Reprinted with permission from the Journal of the American Society of Nephrology [46].

**Figure 4:**
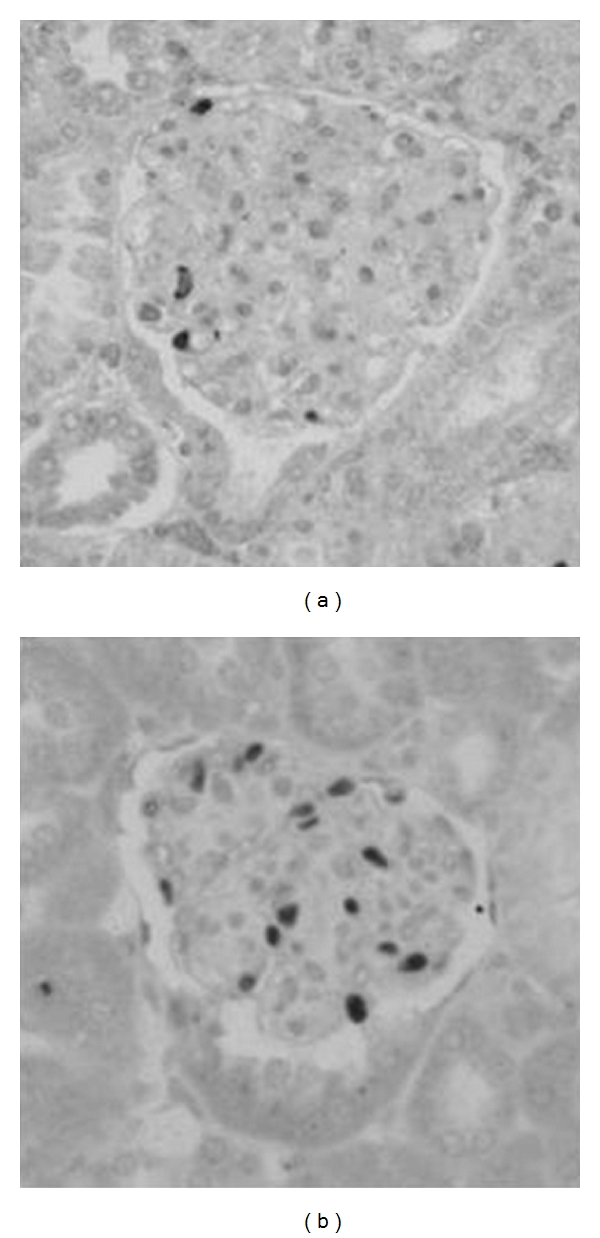
Podocyte loss in BTBR ob/ob mice. BTBR ob/ob mice have reduced podocyte number. (a and b) There is a reduction in podocyte number assessed by WT-1 staining, in BTBR ob/ob (a) compared with BTBR WT (b) mice. Reprinted with permission from the Journal of the American Society of Nephrology [46].

The BTBR ob/ob mouse model of DN comes close to meeting all of the proposed criteria of the AMDCC (albuminuria, pathologic changes) and offers several important advantages compared with existing DN models. The most important of these is the degree to which it supposedly reproduces the essential structural and functional features of human diabetic glomerular injury. Glomerular hypertrophy, marked expansion of mesangial matrix, mesangiolysis, and capillary basement membrane thickening have been identified in this model (Figure 3). Loss of podocytes (Figure 4) is also present in the BTBR ob/ob model [46]. 

The functional consequence of these changes in humans—marked proteinuria—also is present in this mouse model with a 10-fold increase in urinary protein excretion compared with controls [46]. Second, the model is robust and progressive: BTBR ob/ob mice uniformly develop features of DN and do so in a predictable time course in which podocyte loss is already detectable by 8 weeks of age and persists throughout the disease. Significant proteinuria is detectable as early as 8 weeks of age, corresponding with detectable podocyte loss, although it can be detected in some mice at even earlier ages, albeit without achieving statistical significance, when comparing 4-week-old cohorts with controls.

Mesangiolysis is also an early feature of the disease, detectable in approximately 10% of glomeruli at 8 weeks of age and coincides with detectable expansion of the mesangial matrix. These mesangial alterations are progressive with detectable expansion of the mesangial matrix. The BTBR *ob/ob *mouse is among the very few models in which pronounced mesangial expansion and mesangiolysis resembling advanced human DN predictably develop [46]. Third, DN develops more rapidly in BTBR *ob/ob *mice compared with models of leptin receptor deficiency (*db/db *mice) or most other mouse models currently used to study DN [7], which often require from 30 to 50 weeks or more to develop relevant lesions. The relatively rapid onset allows opportunities for testing therapeutic strategies aimed at halting or ameliorating DN in a much shorter time span. BTBR ob/ob mice develop a constellation of abnormalities that closely resemble advanced human DN more rapidly than most other murine models, making this strain particularly attractive for testing therapeutic interventions [46].

### 4.4. GIPR^dn^ Transgenic

Transgenic mice, expressing the mutated human glucose-dependent insulinotropic polypeptide receptor (GIPR), were generated under the control of the rat proinsulin 2 gene promoter in pancreatic beta cells [47]. These GIPR^dn^ transgenic mice exhibit an early disturbance in pancreatic islet development (severe reduction of beta-cell mass, disturbed composition of islets, and decreased islet neogenesis), diminished insulin secretion, and early-onset diabetes mellitus, without obesity or insulin resistance. In type 2 diabetic patients, a major abnormality is reduced insulinotropic action of GIP [48], as well as reduced volume density and mass of  beta cells in the pancreas [47]. Therefore, GIPR^dn^ transgenic mice resemble important aspects of human type 2 diabetes mellitus.

GIPR^dn^ transgenic mice develop progressive diabetes-associated kidney lesions with many parallels to the human disease, that is, renal, glomerular, and podocyte hypertrophy, thickening of the GBM, reduction of the glomerular density of podocytes, progressive glomerulosclerosis albuminuria, and tubulointerstitial changes. This experimental model, may serve as an excellent new model for studying the pathogenesis of DN, the sequence of structural, functional, and molecular changes of glomerular cells, and for testing the efficacy of new therapies [49].

### 4.5. GLUT-1 Transgenic

Heilig's group recently published the phenotype of transgenic GLUT1-overexpressing mice (GT1S). This is an intriguing model designed to characterize the roles of GLUT1 and intracellular glucose in the development of glomerular disease without diabetes [50]. Kidneys of GT1S mice overexpressed GLUT1 in glomerular mesangial cells and small vessels without increased expression in renal tubules. GT1S mice were neither diabetic nor hypertensive. Glomerular GLUT1, glucose uptake, mean capillary diameter, and mean glomerular volume were all increased in the GT1S mice. The transgenic glomeruli revealed diffuse mesangial expansion on light microscopy, occasionally with nodular features, although the nodules were more cellular and do not really replicate the human Kimmelstiel Wilson nodules. Moderately severe glomerulosclerosis (GS) was found by 26 weeks of age in GT1S mice, with increased glomerular Type IV collagen and fibronectin. Modest increases in GBM thickness and albuminuria were detected with podocyte foot processes largely preserved, in the absence of podocyte GLUT1 overexpression [50]. Activation of glomerular PKC, along with increased TGF*β*1, VEGFR1, VEGFR2, and VEGF was all detected in glomeruli of GT1S mice, likely contributing to GS. The transcription factor NF-*κ*B was also activated. Overexpression of glomerular GLUT1, mimicking the diabetic GLUT1 response, produced numerous features typical of diabetic glomerular disease, without diabetes or hypertension. This suggested that the GLUT-1 transporter overactivity plays an important role in the development of DN possibly by locally increasing glucose uptake at the glomerular level. In this respect, this model offers a strong evidence for intracellular glucose in the development of glomerulosclerosis [50]. 

### 4.6. PodIR Knockout

To determine whether insulin signaling in podocytes affects glomerular function in vivo, Welsh's group generated mice with specific deletion of the insulin receptor from their podocytes (podIRKO). PodIRKO mice were generated by crossing floxed insulin receptor mice with podocyte-specific Cre recombinase mice driven by both the nephrin and podocin promoters [51, 52]. Detailed renal evaluation at 3 weeks of age was normal in podIRKO mice with no abnormality identifiable using either light or electron microscopic analysis. At 5 weeks of age, both podIRKO models (nephrin and podocin promoter) started to develop albuminuria accompanied with loss of the podocyte foot process structure detected by electron microscopy [52]. At 8 weeks of age, significant levels of albuminuria and histological changes such as extensive loss of foot process structure and podocyte apoptosis were present in podIRKO. Furthermore, at 13 weeks of age, podIRKO mice had increased amounts of glomerular matrix, thickened GBM, and increased levels of glomerulosclerosis [52]. As they aged, these pathological features became more prominent, with some of the podIRKO mice developing macroscopically shrunken and sclerosed kidneys. These animals develop significant albuminuria together with histological features that recapitulate DN, but in a normoglycemic environment. These novel findings reveal the critical importance of podocyte insulin sensitivity for kidney function [52].

## 5. Genetic Background as a Modifier of Diabetic Kidney Disease

Gurley and others have emphasized the importance of the genetic background in kidney disease development in diabetic mice [7]. Their initial studies focus on STZ-induced diabetes but they are relevant to most models of diabetes. They reported among five common inbred mouse strains a hierarchical response of blood glucose levels to STZ-induced diabetes (DBA/2>C57BL/6>MRL/Mp>129/SvEv>BALB/c). In all five strains, males demonstrated much more robust hyperglycemia with STZ than females. STZ-induced diabetes was associated with modest levels of albuminuria in all of the strains, but was greatest in the DBA/2 strain, which also had the most marked hyperglycemia [53]. Observed renal structural differences between the strains were limited to mesangial expansion, but the strong correlation between high blood glucose and mesangial size expansion suggests that the size differences were caused by the differences in blood glucose levels. The differences in the responses to STZ-induced diabetes suggest that DBA/2 is the most susceptible to diabetic nephropathy and is most likely the most useful platform for model development [53]. It is important to emphasize the relative resistance to diabetic nephropathy of the widely used C57BL/6 mouse [7]. The Mouse Phenome Database is a useful source for comparative data of basal metabolic parameters distinguishing the more commonly used inbred strains (http://phenome.jax.org/). This website contains albumin-creatinine ratio data for males and females of 30 inbred strains [7].

## 6. Conclusions

The use of experimental animal models of DN has provided valuable information regarding some aspects of DN. Classical experimental models of type 2 DN have been available. However, for some time these models have not been able to reproduce features of advanced DN such as nodules in the glomerular tuft or glomerulosclerosis. Moreover, information on progressive decline in GFR is often missing in these models. In particular, through the efforts of the AMDCC investigators as well as others, using genetic breeding and other means to enhance disease severity, the characteristic features of experimental diabetic nephropathy are becoming more apparent. The importance of genetic background on susceptibility and resistance to kidney disease is increasingly recognized as a key factor. Data from the Diabetic Complications Consortium group has been very helpful for the researchers by creating a website that includes a protocol for validation of mouse models of diabetic nephropathy and updated characterization of different diabetic models.

## Figures and Tables

**Table 1:** Some new models studied for diabetic nephropathy.

Animal model	Strain	Kidney pathology	References
Db/db eNOS^−/−^	BKS	Significant albuminuria, decreased GFR, markedly increased mesangial matrix expansion, glomerular basement membrane thickening, arteriolar hyalinosis, mesangiolysis, nodular glomerulosclerosis, and tubulointerstitial injury	Zhao et al. [25] and Mohan et al. [41]

NONcNZO10/LtJ	NON/LtJ + NZO/HlLt	Albuminuria, glomerulosclerosis, intraglomerular capillary thrombi and lipid deposition, nephritis, and Ig deposition	Reifsnyder and Leiter [29, 30]

BTBR^ob/ob^	BTBR	Albuminuria, loss of podocytes, extensive mesangial expansion, mesangiolysis, basement membrane thickening, and interstitial fibrosis	Hudkins et al. [46]

GIPR^dn^ transgenic	CD1	Renal, podocyte and glomerular hypertrophy, mesangial expansion, and matrix accumulation, glomerulosclerosis, proteinuria, and tubulointerstitial lesions	Herbach et al. [49]

GLUT1 transgenic	C57BL6/J	Albuminuria, glomerular hypertrophy, mesangial expansion, and glomerulosclerosis	Wang et al. [50]

podIR knockout (podocin or nephrin promoter)	Mixed genetic background (C57BL/6, 129/SV, and FVB)	Albuminuria, loss of foot process structure, podocyte apoptosis, increased glomerular matrix, thickened GBM, glomerulosclerosis, and kidney sclerosis	Welsh et al. [52]
